# From Retinal Waves to Activity-Dependent Retinogeniculate Map Development

**DOI:** 10.1371/journal.pone.0031553

**Published:** 2012-02-28

**Authors:** Jeffrey Markowitz, Yongqiang Cao, Stephen Grossberg

**Affiliations:** 1 Center for Adaptive Systems, Department of Cognitive and Neural Systems, Boston University, Boston, Massachusetts, United States of America; 2 Center for Excellence for Learning in Education, Science and Technology Boston University, Boston, Massachusetts, United States of America; University of Pittsburgh, United States of America

## Abstract

A neural model is described of how spontaneous retinal waves are formed in infant mammals, and how these waves organize activity-dependent development of a topographic map in the lateral geniculate nucleus, with connections from each eye segregated into separate anatomical layers. The model simulates the spontaneous behavior of starburst amacrine cells and retinal ganglion cells during the production of retinal waves during the first few weeks of mammalian postnatal development. It proposes how excitatory and inhibitory mechanisms within individual cells, such as Ca^2+^-activated K^+^ channels, and cAMP currents and signaling cascades, can modulate the spatiotemporal dynamics of waves, notably by controlling the after-hyperpolarization currents of starburst amacrine cells. Given the critical role of the geniculate map in the development of visual cortex, these results provide a foundation for analyzing the temporal dynamics whereby the visual cortex itself develops.

## Introduction

**Figure 1:**
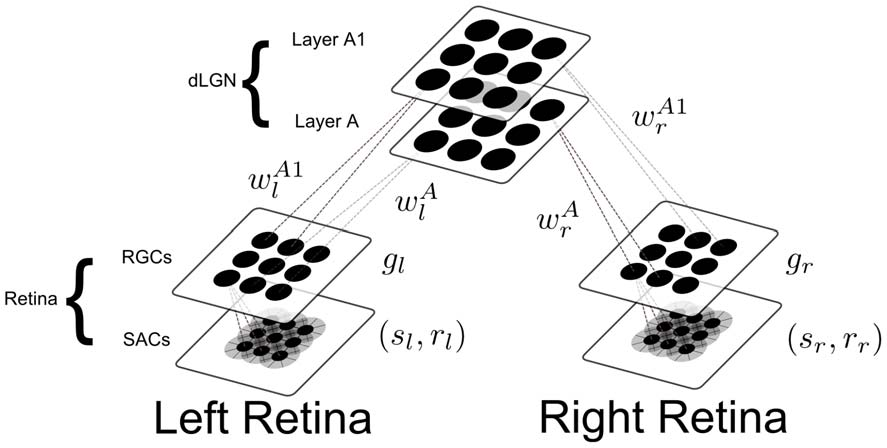
A diagram of the full model circuit.

This article describes a neural model of how spontaneous retinal waves are formed in infant mammals, and how these waves organize activity-dependent development of a topographic map in the lateral geniculate nucleus, or LGN, with connections from each eye segregated into separate anatomical layers of the LGN (Figure 1). Recent work in imaging the retina through multi-electrode arrays [1] and fluorescent dyes [2] before eye opening in mammals, and some invertebrates, has cast new light on how these spontaneous traveling waves in the retina are formed. While their existence has been known for over two decades [3], experimentalists have just begun to untangle their molecular basis and functional implications [4]–[11]. Our model builds upon previous theoretical work [2], [12]–[14] to clarify key properties of these waves and to show how these properties may function to organize the development of the retinogeniculate map. The model includes two key cell types: starburst amacrine cells (SACs) and retinal ganglion cells (RGCs) that interact through time.

Albeit simple, the new model goes beyond earlier modeling efforts by explicitly accounting for the role of intracellular mechanisms such as Ca^2+^-activated K^+^ channels, cAMP, and afterhyperpolarization currents (AHPs) in controlling spatiotemporal properties of retinal waves [5]. These results include novel predictions concerning how these intracellular mechanisms regulate retinal waves. For example, the model predicts that decreasing the strength of AHPs, formulated here as an activity-dependent recovery process (see equation (7)), increases the frequency and velocity of the waves generated by SACs, and vice versa for increasing the strength of AHPs. Thus, our model advances current knowledge by simulating how single-cell currents may give rise to emergent dynamical network properties, such as wave velocity, shape, and periodicity.

Retinal waves cause spontaneous bursts of action potentials in RGCs, on the order of 10–100 Hz, that move in a wave-like fashion across 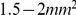 sections of the retina in the first two weeks of postnatal development, just prior to eye opening in cat, ferret, and mouse. There are two stages of retinal waves, normally classified into early (<P8) and late (>P8) stage waves. This article concerns early stage waves only, which have been more fully characterized as spatially correlated patterns of activity that travel across the RGC network. Blockade of nicotinic acetylcholine receptors (nAChRs) in the retina leads to the disruption of waves, as well as of the development of the early visual system [8] (although see [15]). The mature organization of the mammalian LGN thus appears to be both caused [16] and maintained [17] by spontaneous retinal activity. The current model simulates how the retinal waves can organize retinogeniculate map development, leading to a learned map in which each eye activates a different layer (A or A1) in the LGN. Moreover, retinal waves also may play a role in the early formation of receptive field properties and ocular dominance columns in V1 [18].

## Materials and Methods

### Model Retina

As noted above, the model (Figure 1) incorporates interacting SACs and RGCs, in keeping with experimental data [15] and the structure of previous models [2], [14] about retinal waves. Model SACs occupy the first layer, where they are laterally connected to each other with isotropic distance-dependent Gaussian weighting functions. Anatomical data estimate their effective input radius at approximately 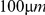 [9]. This lateral communication approximates the function of transmission via nicotinic acetylcholine receptors (nAChRs) during early development, which are thought to be a critical mediator of waves and thereby retinogeniculate development [8], [19] Spontaneous activity within the SACs is generated using a Poisson process.

All model cells are point neurons whose single compartment voltage, 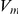, obeys:
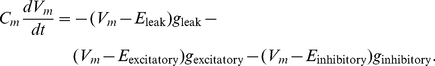
(1)In (1), 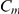 is the membrane capacitance; 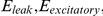 and 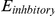 are the three Nernst, or reversal potentials; and 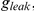 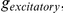 and 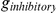 are the three intracellular conductances. Setting 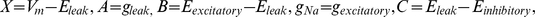 and 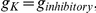 (1) becomes:
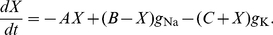
(2)In this notation, 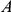 is the passive decay rate, 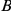 is maximum value of potential 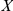, and 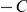 is the minimum value of 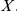


### Starburst Amacrine Cells

The activities 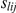 and 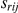 of SAC cells 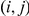 in the left and right eyes obey:
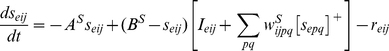
(3)respectively. As in (2), parameter 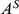 in (3) is the decay rate; 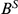 is the excitatory saturation point; 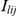 and 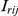 are endogenously active inputs that are defined by a Poisson distribution:
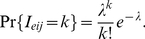
(4)In (3), subscript *l* denotes the left eye and *r* the right eye. In the Poisson distribution in (4) that defines the probabilistic input term 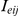, Pr denotes the probability, and 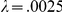. The excitatory recurrent interactions 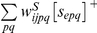 in (3) between SAC cells are mediated by Gaussian connection weights across space:
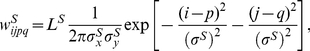
(5)where 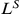 is a scaling factor, and 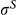 defines the variance along the x and y axes, which are assumed to be equal, as suggested by data from imaging of SACs [9]. The recurrent signal function in (3) is a half-wave rectified function of cell activity; e.g.,
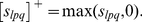
(6)In (3), the inhibitory shunting term in (2) is missing, since GABAergic synapses have been shown to be excitatory in the retina during the presence of spontaneous waves [9], which could be due to changes in the intracellular concentration of chloride [20]. More precisely, if the intracellular concentration of chloride were increased until it exceeded the extracellular concentration, then chloride ions would flow out of the cell upon receptor activation via GABA.

Lastly, terms 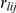 and 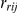 in (3) are intracellular processes that govern each SAC cell's refractory period via afterhyperpolarization, or AHP, currents:
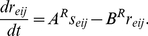
(7)In (7), 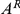 is the rate at which each 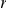 increases, analogous to intracellular mechanisms that lead to inhibition as a result of spiking; e.g. Ca^2+^-activated K^+^ channels [21]. By the same token, 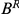 defines the rate with which each 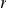 decreases, thereby upregulating the excitability of each SAC, similar to the role of cAMP and its correspondent signaling cascades [5], [22]. The variable 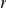 is similar to the recovery variable used in the Fitzhugh-Nagumo equation to describe the spiking rate of a single neuron [23]. It describes the overall state of activity-dependent intracellular processes that modulate a cell's firing rate. The variable 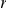 increases when the cell begins to fire, and it steadily decreases once the recovery variable has sufficiently suppressed the firing rate. For example, an increase in the SAC cell activity 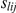 in (3) leads to an increase in 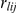 in (7) that, in turn, suppresses 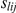 via (3), leading to a subsequent decrease in 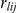 until 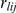 can no longer suppress 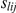. This interaction qualitatively simulates the recruitment of Ca^2+^-activated K^+^ channels, which have been shown to control the AHPs of SACs using *in vitro* whole cell patch clamp [24]. More precisely, an increase in a cell's firing rate leads to an increase in the intracellular concentration of Ca^2+^ which, in turn, opens Ca^2+^-activated K^+^ channels that act to electrically shunt the cell by increasing K^+^ conductance on a slow timescale, decreasing the membrane resistance for tens of seconds. It also appears that these channels are voltage-independent (similar to the small conductance, or SK, channels) and can only be opened by an increase in cytosolic Ca^2+^ [24], although cytosolic Ca^2+^ can be affected by the conductance of voltage-gated Ca^2+^ channels.

### Retinal Ganglion Cells

The SACs send half-wave rectified signals to the RGCs, which smooth SACs activity through leaky integration. The activities 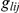 and 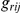 of RGCs in position 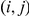 in the left (*l*) and right (*r*) eyes obey:
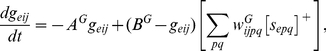
(8)respectively, where 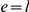 or 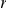. In (2), the weights 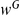 are Gaussian functions of distance with the same spatial parameters as in (5). Finally, the RGC output signals to the LGN are thresholded at a positive threshold 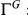


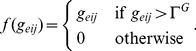
(9)By (9), model RGCs act as a set of thresholded leaky integrators that remove noise from the SAC layer–that is, the RGCs only relay activity at a sufficiently high firing rate–similar to the model in [2]. Supporting this assumption, experimental data have demonstrated that, outside of bursting related to waves, RGC activity may not be tightly coupled to SAC activity [24]. See Table 1 for the parameters for the model retina.

**Table 1:** Parameters used for model simulations.

Parameter	Value	Meaning
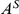	6.5	SAC leak
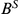	5	SAC maximum excitability
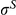	1	SAC dendritic spread
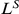	3	Weight scaling
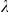	.0025	Parameter for Poisson process
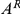	8	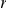 growth rate
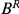	.09	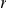 decay rate
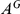	10	RGC leak
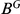	50	RGC maximum excitability
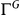	3	RGC output threshold
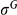	1	RGC dendritic spread
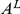	5	dLGN leak
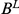	5	dLGN maximum excitability
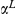	11	Retinotopic bias
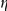	7	Noise in retinogeniculate axons
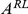	1e-5	Learning rate
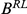	1	growth factor

### Lateral Geniculate Nucleus Cells

The RGC outputs activate cells in the dorsal LGN (dLGN) in layers 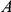 and 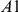 whose activities 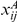 and 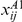 at position 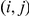 obey the equations:
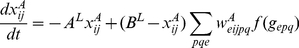
(10)and
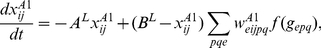
(11)respectively. The adaptive weights 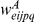 and 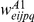 in (10) and (11) from the RGC at position 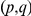 and eye 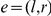 to the dLGN cell at position 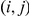 and layer 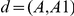 are initially chosen at time 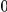 to obey the equations:
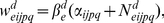
(12)where
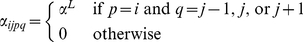
(13)and
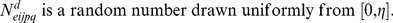
(14)This defines a noisy topography where arborization from retinogeniculate axons undershoots and overshoots its target along the dorsoventral axis in early development [25], [26]. The eye-specific bias terms 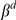 in (12) multiply the afferents from the left eye to layer A and the right eye to layer A1 by an eye-specific scaling factor with parameter 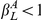 (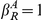) for the projections from the left (right) eye to layer A and 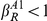 (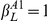) for projections from the right (left) eye to layer A1. As a result, afferents from both eyes contact both layers of the LGN, albeit with a slight bias to the eye that eventually dominates, prior to the critical stage of mammalian retinogeniculate development of eye-specific layers [26], [27].

The activity-dependent release of learning-inducing neurotrophins from dLGN cells is modeled by sigmoid signals of LGN activities:
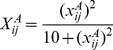
(15)and
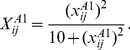
(16)


### Retinogeniculate Map Learning

Learning in the adaptive weights 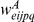 and 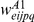 in equations (10) and (11), from the eyes 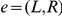 to the LGN layers 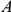 and, 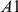 is gated on and off by these neurotrophic signals, and obeys the following equations:
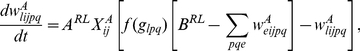
(17)

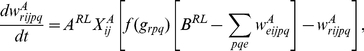
(18)

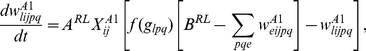
(19)and
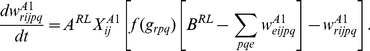
(20)Equations (17)–(20) define a self-normalizing *instar* learning law [28]–[30] wherein 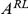 is a learning rate and 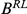 a limited growth factor released by coincident LGN bursting (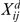), where *d* equals *A* or *A1*, and RGC bursting (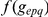). Learning and forgetting are both gated by postsynaptic activity (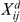). This process could be carried out through the use of a limited growth factor, such as BDNF or NT-4 [31], [32], when there is a coincident rise in the firing rates of RGCs and dLGN cells [33], [34]. Competition for a limited neurotrophin is shown herein to cause bounded synaptic growth. It allows for the eye with a slight initial advantage to dominate a given layer of the dLGN. In particular, as weights with a slight bias grow, they prevent the growth of other competing weights.

Models that rely on inter-synaptic competition have used explicit divisive or subtractive normalization to achieve similar results [13], [35]. These latter approaches are not biologically realistic because it is unclear how synapses would implement global divisive normalization. The learning equations (17)–(20) can replicate map formation by using local interactions that are consistent with what is known about how synapses in the developing retinogeniculate projection compete; in particular, when a postsynaptic cell becomes active, its abutting weights can compete for growth. The parameters for the model retinogeniculate pathway are given in Table 1.

## Results

### Retinal Wave Simulations

**Figure 2:**
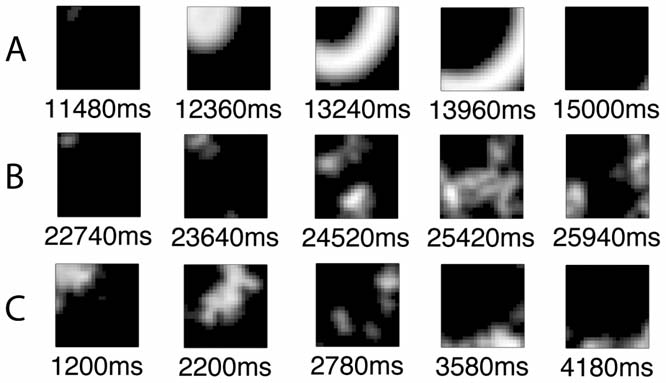
Depiction of a model wave generated using the parameters given in **Table 1** (A) and through randomizing SAC refractory periods (B and C). Model RGCs are shown using an intensity plot where the brightness scales with the firing rate. The waves propagate in a radially symmetric pattern due to the isotropy of the SAC lateral weights, or model dendrites. Other geometries were achieved through randomizing 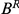 by choosing 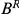 from a normal distribution centered on .09 where 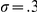 (negative values were set to zero).

Retinal waves [10] were simulated in order to tease apart the effects of excitatory and inhibitory intracellular mechanisms; e.g., the cAMP and Ca^2+^ currents that are represented in (7). These intracellular mechanisms are assumed to control the AHP of SACs that modulate wave velocity and periodicity. The model replicates the general spatiotemporal properties of waves observed *in vitro* in a number of animals, including mice and ferrets. A sample wave is depicted in Figure 2. The properties most commonly observed and quantified are the inter-wave interval (IWI), or the time between waves, and the wave velocity [1], [2], [5]. These properties were the focus of the simulations. Wave events were identified by determining if the number of active cells at a given time point exceeded a predetermined threshold, similar to a method used to quantify neuronal avalanches [36], which are neural events quite similar to retinal waves. The movement of the center of mass (CoM) of cell activity across time was used to measure wave velocity [14], where the CoM for each retina e is defined by:
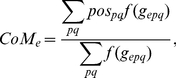
(21)where 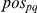 denotes position (p, q) and the RGC outputs 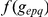 are defined in (9).

First, model simulations were compared to Ca^2+^-imaging data from [2] that were corroborated using a 512 electrode array [1]. The model was numerically integrated for 40000 seconds (approximately 11 hours) using Euler's method with a time step of 20 ms, and the output of the RGC layer was stored for subsequent analysis. Since the model parameters could not be directly fit to biophysical data (e.g., the batch concentration of forskolin, which increases the intracellular concentration of cAMP), they were determined empirically.

In all the retinal simulations, waves were detected by counting the number of active cells at each time step and comparing with a pre-determined threshold. The algorithm assumes that the beginning and end of a wave are marked by crossing the threshold of 10 cells going active. Thus, if more than ten cells (out of 576) burst, a wave begins, and the wave ends as soon as less than ten cells are bursting. Each time step in between these two events is considered part of a single wave. This algorithm eased the significant computational load of analyzing the simulation results (24×24 values over 200000 time steps). Hundreds of identified target waves were checked by eye to ensure the validity of the algorithm.

**Figure 3:**
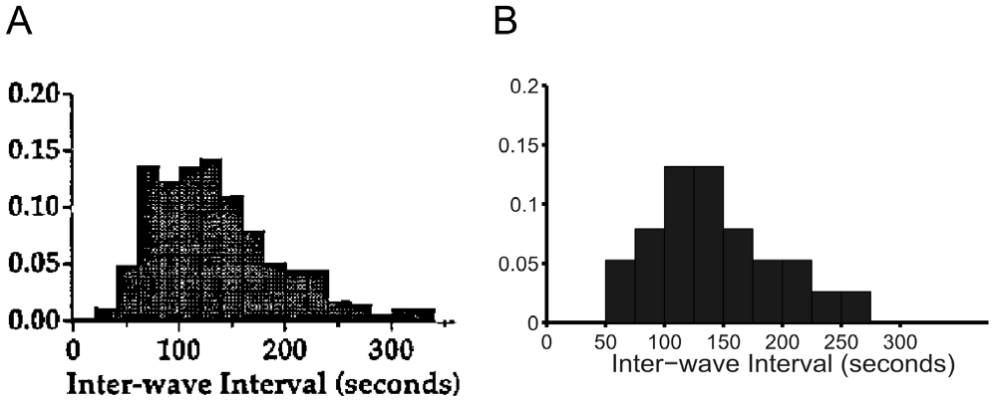
Inter-wave interval (IWI) distributions from Ca^2+^-imaging data (A) and the model simulation (B). A comparison the IWI distributions from model simulations with Ca^2+^-imaging data [2]. The Ca^2+^-imaging data is derived from multiple retinas. Likewise, the simulation results are an aggregate of multiple simulations that used different values of 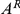 (6,6.15,6.3,6.45,6.6,6.75,6.9,7.05). [Experimental data in (A) are reproduced with permission from [2].]

**Figure 4:**
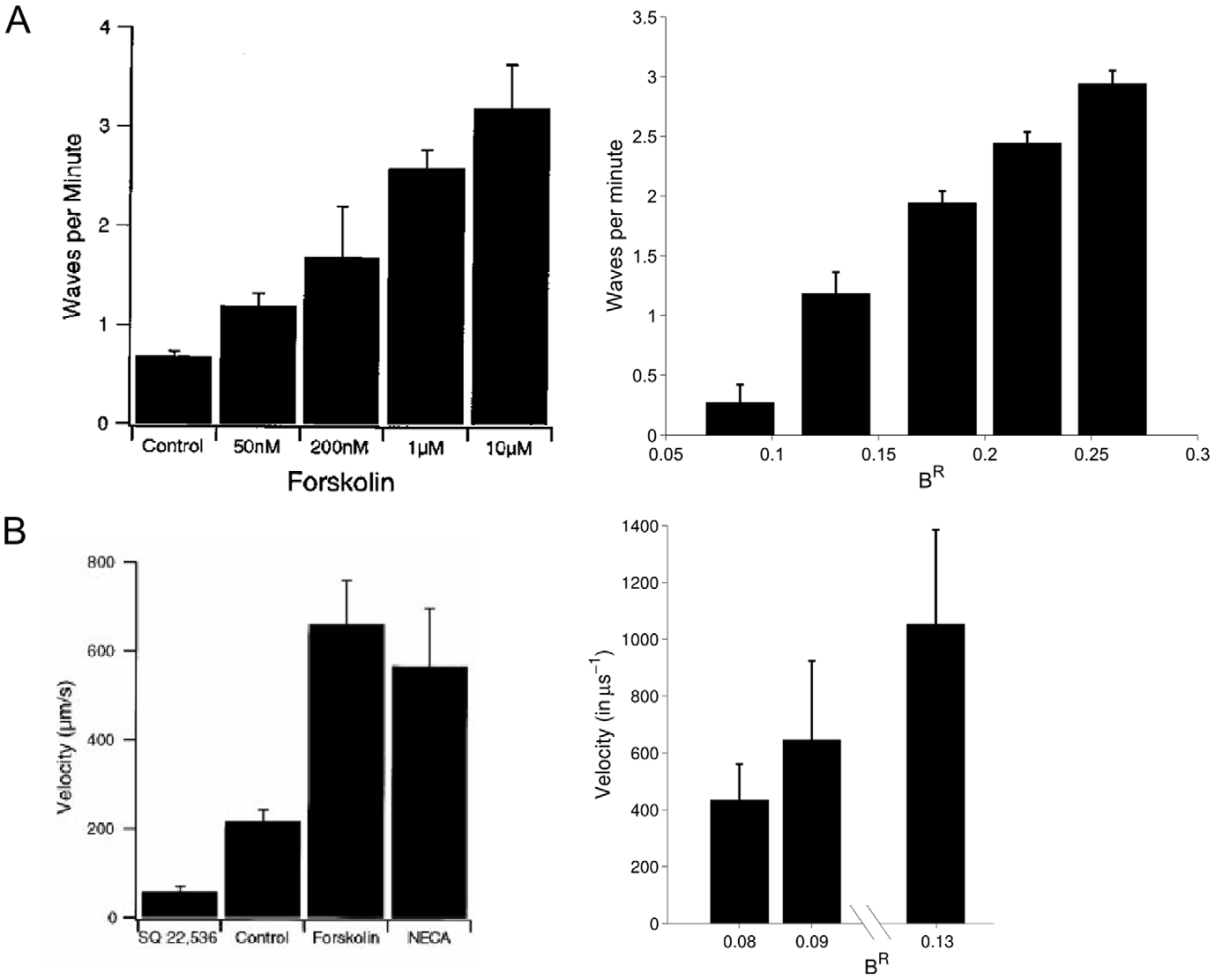
Comparison of waves per minute (A), and velocity (B), between simulations while increasing 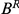
 **in (8), a parameter analogous to excitatory intracellular mechanisms (e.g. cAMP-related signaling cascades) in experiments conducted under various levels of cAMP.** In (A), the average waves per minute (WPM) is compared between the model (right) and an experiment (left) under forskolin treatment, an agonist of cAMP [5]. Here, WPM monotonically increases as a function of 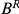, similar to the forskolin treatment *in vitro*. In (B), the average velocity is given for model simulations (right) and cAMP perturbations in the same experiment (left). Generally, velocity increases with 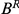 in the model, which is also observed under pharmacological manipulation of cAMP levels. For the model, the mean movement of the centroid of the wave across time is used as a measure of velocity, as in (25).

**Figure 5:**
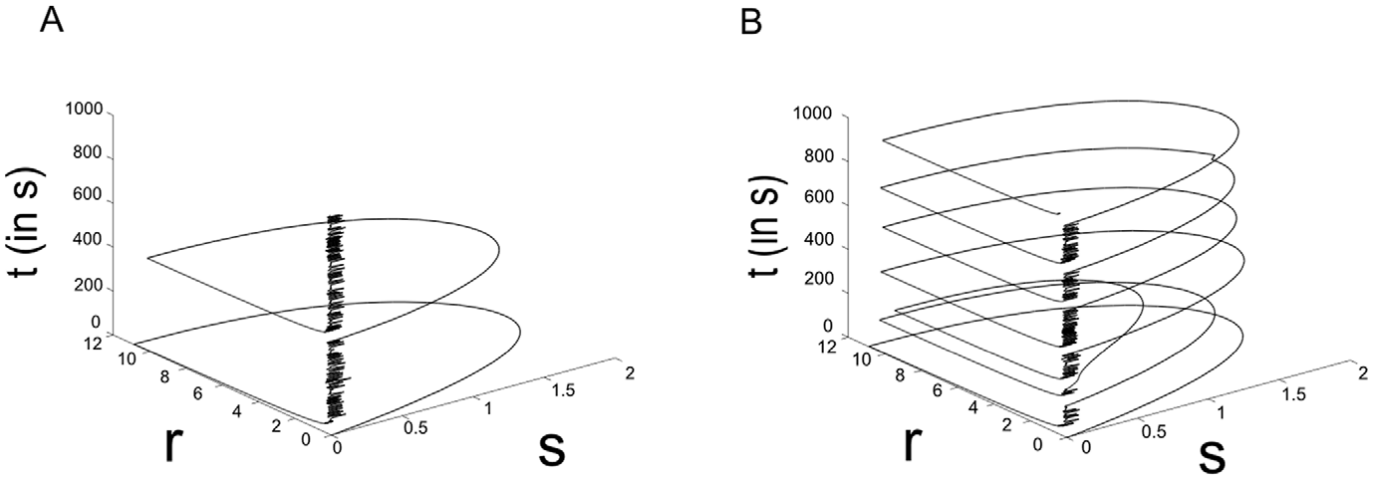
Single cell dynamics of a model SAC when setting 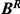
 **in (8) low (A), and high (B), to .09 and .095, respectively.** Shown are the dynamics of a single cell in a full simulation of the model retina over 1000 seconds. Here, 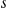 is the firing rate of the model SAC, and 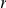 the amount of inhibition due to intracellular processes. By setting 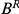 high, the decay rate of inhibition decreases, analogous to the application of forskolin to acute retinal slices. This has the effect of increasing both the WPM and the wave velocity (see Figure 4).

Figure 3 shows a good qualitative match of IWI distributions between the model and experiment. Increasing parameter 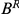 in equation (7), analogous to increasing the level of intracellular cAMP or other intracellular excitatory mechanisms, decreases AHPs in model SACs, and thereby shifts both the IWI and velocity distributions to the right (Figure 4). Additionally, the single cell dynamics of model SACs is significantly altered; SAC cells enter an active, or bursting state (i.e., a state of sustained high firing rate) more frequently (Figure 5). Thus, model SACs control IWI, velocity, and SAC firing characteristics using activity-dependent intracellular processes.

**Figure 6:**
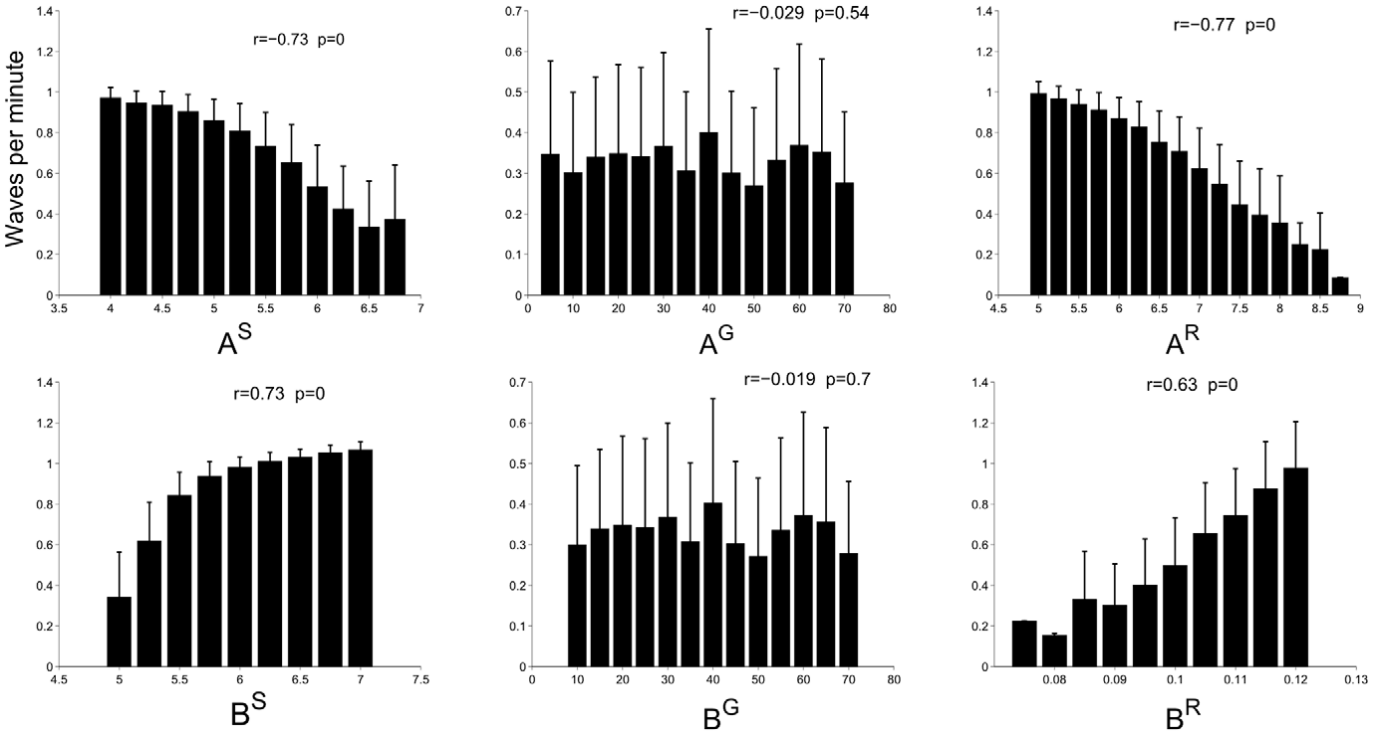
The effect of changing model parameters on waves per minute (WPM). The effects on WPM of varying the following parameters were studied: leak 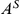 and maximum excitability 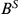 in model starburst amacrine cells (SACs)–see (3); similar parameters 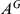 and 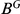 in model retinal ganglion cells (RGCs)– see (10); and other intracellular excitatory 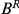 and inhibitory 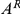 mechanisms in the AHP currents–see (8). Increasing 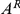 or 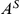 decreases WPM, while increasing 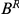 or 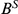 increases WPM. The parameters 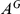 and 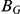 governing the behavior of model RGCs have no significant effect on WPM, indicating that SAC dynamics alter WPM. Means of WPM across multiple simulations are shown, with error bars indicating the standard deviation.

**Figure 7:**
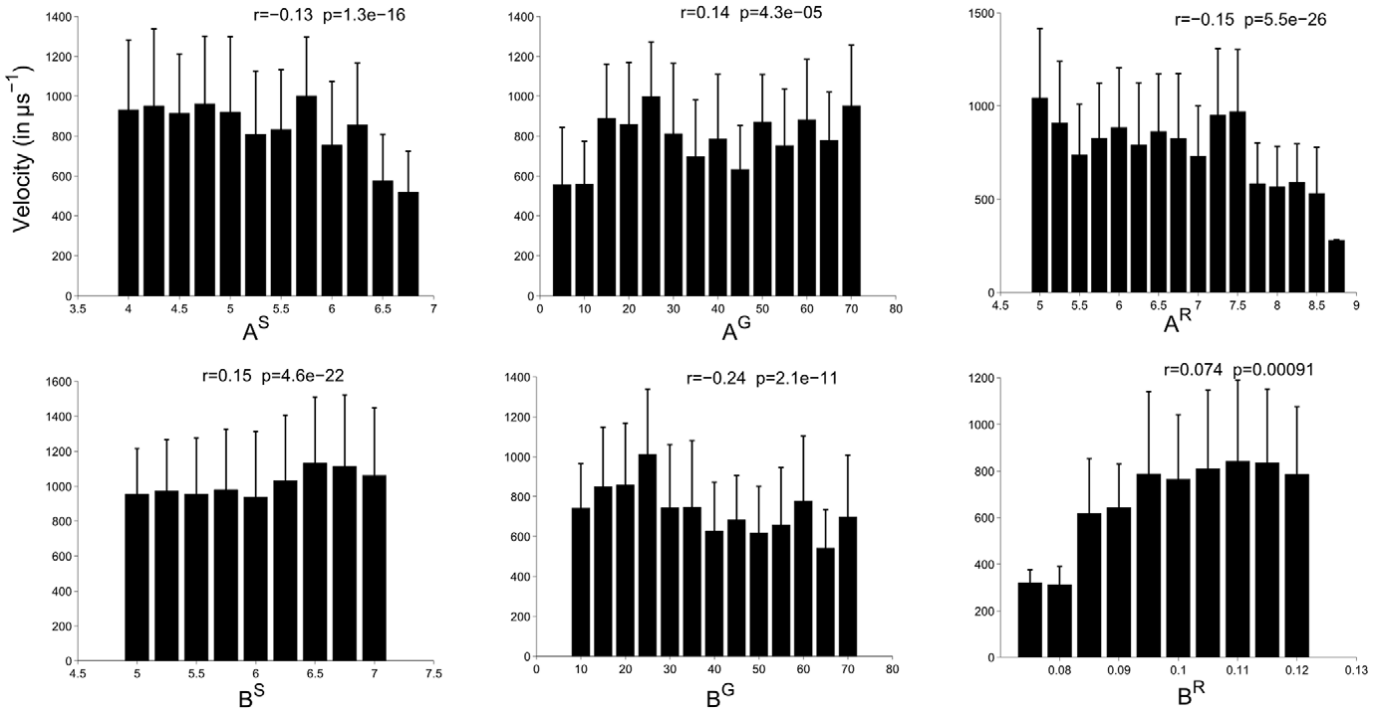
The effect of changing model parameters 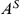
 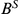 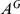 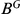 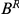 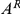 **on wave velocity.** The effects on velocity are weak, but statistically significant. As with WPM, increasing 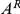 and 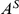 decreases velocity, and vice versa for 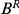 and 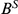. Unlike the periodicity of waves, their velocity is significantly modulated by the properties of the model RGCs via 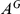 and 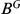.

To more comprehensively understand how model parameters affect the IWI and velocity, simulations systematically explored the parameter space (see Figures 6 and 7). The results demonstrate that the inhibitory and excitatory terms in the two SAC equations modulate waves in complementary ways: Increasing the SAC decay rate 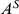 and the excitatory saturation potential 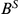 in (3) decrease and increase waves per minute (WPM, inversely proportional to IWI) and wave velocity, respectively, and similarly for 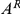 and 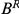 in (7). These results predict that upregulating the decay rate or intracellular inhibitory mechanisms (e.g., cytoplasmic Ca^2+^ concentration) of SACs should decrease wave velocity and WPM, while increasing maximum excitability (e.g., density of voltage-gated Na^+^ channels) or intracellular excitatory mechanisms (e.g., intracellular cAMP concentration) should increase velocity and WPM. Additionally, these simulations show a significant relationship between velocity and WPM, which has been observed in experiments [5] and other theoretical work [2].

Whereas all SAC parameters influence wave periodicity and velocity, the RGC parameters 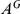 and 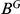 only affected wave velocity. This is due to the fast-slow dynamics present in the SACs and the assumption that RGCs merely filter SAC activity en route to the dLGN.

### Retinogeniculate Map Development Simulations

Simulations were carried out to demonstrate that waves generated within the model retina can drive the development of a retinogeniculate map whose connections from each eye are segregated into distinct LGN layers A and A1 [37], [38] (see equations (10)–(20)), Molecular guidance cues, e.g. EphA and EphB [39], presumptively form the initial topography of the retinogeniculate projection while a second stage of activity-dependent refinement prunes ectopic axonal arbors (i.e., arbors that resulted in a breakdown of retinotopy) [25], [40], [41]. The model retinogeniculate pathway focuses on this second stage of development.

**Figure 8:**
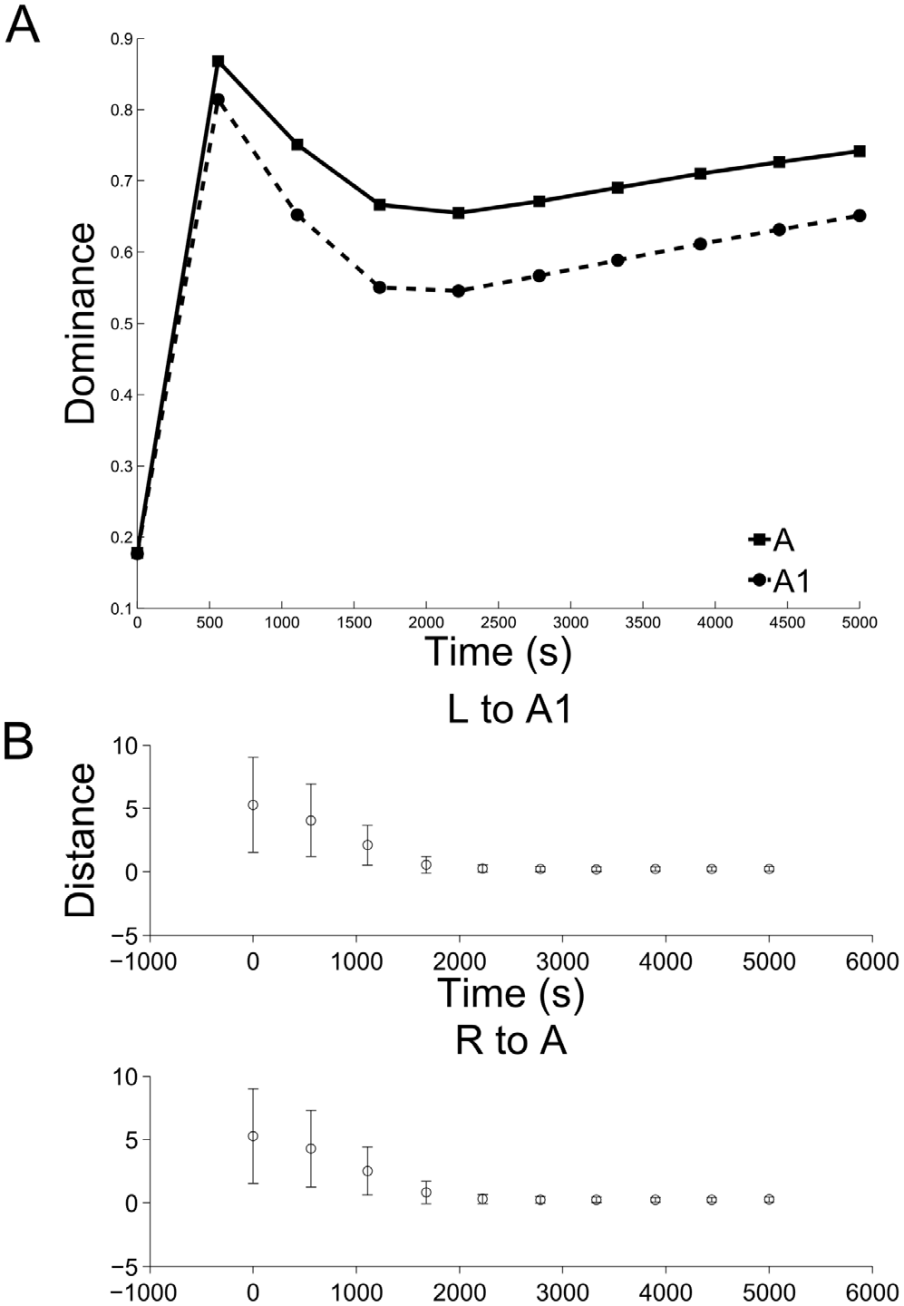
Simulation of retinogeniculate development. (A) The normalized dominance of the initially biased eye grows. For both layers A and A1, the eye that is initiated with weights slighter larger than the other comes to ‘dominate’ that layer over the course of 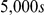 of waves (see (22)). (B) Additionally, the mean distance of the CoM from an ideal CoM for the retinogeniculate weights decreases, indicating that the weights projecting to each dLGN cell become increasingly refined; in particular, ectopic projections are pruned away.

**Figure 9:**
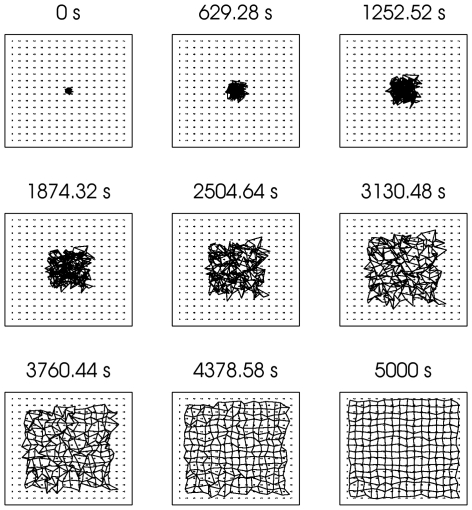
Change in CoM for the model retinogeniculate pathway (from the left retina to layer A1 of the dLGN, both layers develop simultaneously but all results are shown in one pathway for convenience). Using the method of visualizing retinogeniculate weights from [34], the CoM of the weights projecting to a given dLGN cell (see (23)) is depicted as a node connected to its 4 nearest neighbors in solid black (2 for cells at the edge, since the boundaries are non-periodic). The ideal topography is visualized by the dotted grid defined by the points in 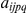, where 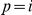 and 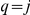 (site of initial retinotopic bias). Over the course of the simulation, the CoMs approach the ideal (the time during the simulation at which the CoMs were assessed is shown just above each figure).

The simulations demonstrate that retinal waves can drive the development of eye-specificity in dLGN layers (Figure 8) as well as refinement of dLGN topography in a single hemisphere (Figure 9). To measure eye-specificity, a normalized measure of the difference in total magnitude (DOM) between the weights projecting from each eye is used:
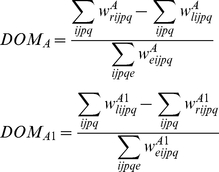
(22)Then, topography was assayed as the summed Euclidean distance between the center-of-mass (CoM) of the weights from the RGC at position (p,q) and projecting to all dLGN cells (i,j) in layers A and A1, respectively:
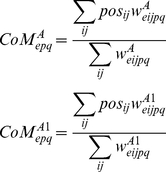
(23)and its correct retinotopic position (i = p, j = q) (see Figure 9B).

First, development of the retinogeniculate pathway was simulated for 5,000 seconds (simulated time) using the standard parameters to generate retinal waves (see Table 1), and each wave was distributed between the retinas such that only one retina was active at a time and consecutive waves occurred in different retinas. Under these conditions, the retinas that were slightly biased to project to each dLGN layer (left retina to dLGN layer A1 and the right retina to dLGN layer A) quickly dominated the retinogeniculate projections at the expense of the opposite retina, indicated by the sharp rise in DOM for layers A and A1 in the first 500 seconds of the simulation (see Figure 8). At the same time, yet on a much slower timescale, the topography of the dLGN was refined (see Figure 9). Thus, the learning rule used in the model retinogeniculate pathway accounts for the development of both layer-specificity and a refined topography.

**Figure 10:**
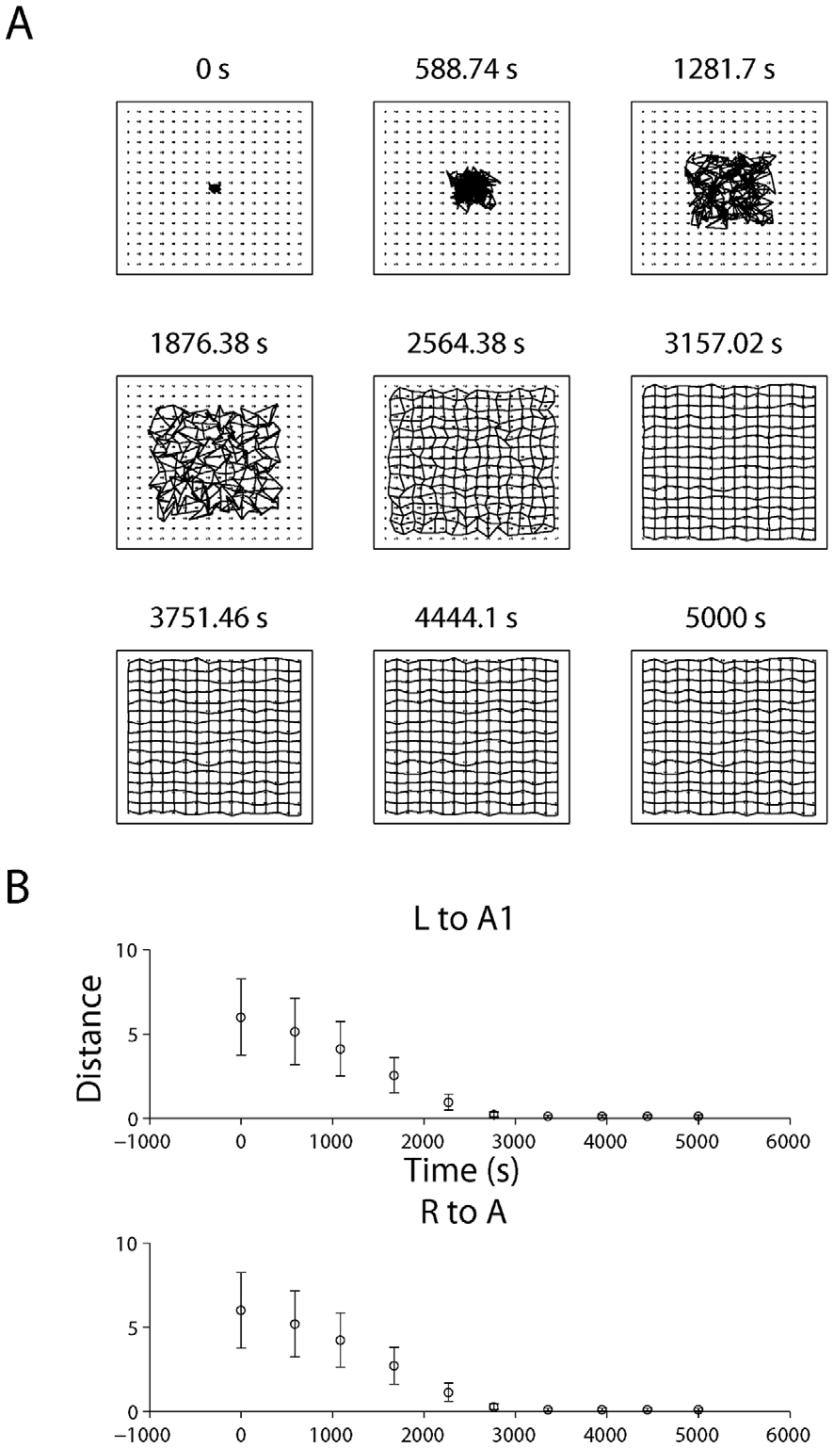
Retinogeniculate development accelerates as wave velocity and wave frequency increases by increasing 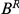
 **(see** **Figure 4** **).** (A) The CoMs approach the ideal grid at a faster pace with accelerated waves, also shown by the sharper decrease in the mean distance of the CoM from their targets (B).

Waves were also generated using different sets of parameters to simulate perturbations to the retina and their effect on retinogeniculate development. First, 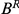 (see (7)) was increased from .12 to .2 to generate faster waves with smaller inter-wave interval distributions (see Figures 3 and 4). This sped up the formation of layer-specificity and, more dramatically, the topography refinement proportional to the increase in 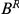 (see Figure 10). When 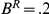, the topography refinement is sped up dramatically, with a noticeable increase in the speed of the initial rise in DOM for both layers. These parameter settings are analogous to the use of forskolin to accelerate waves *in vitro* [5].

**Figure 11:**
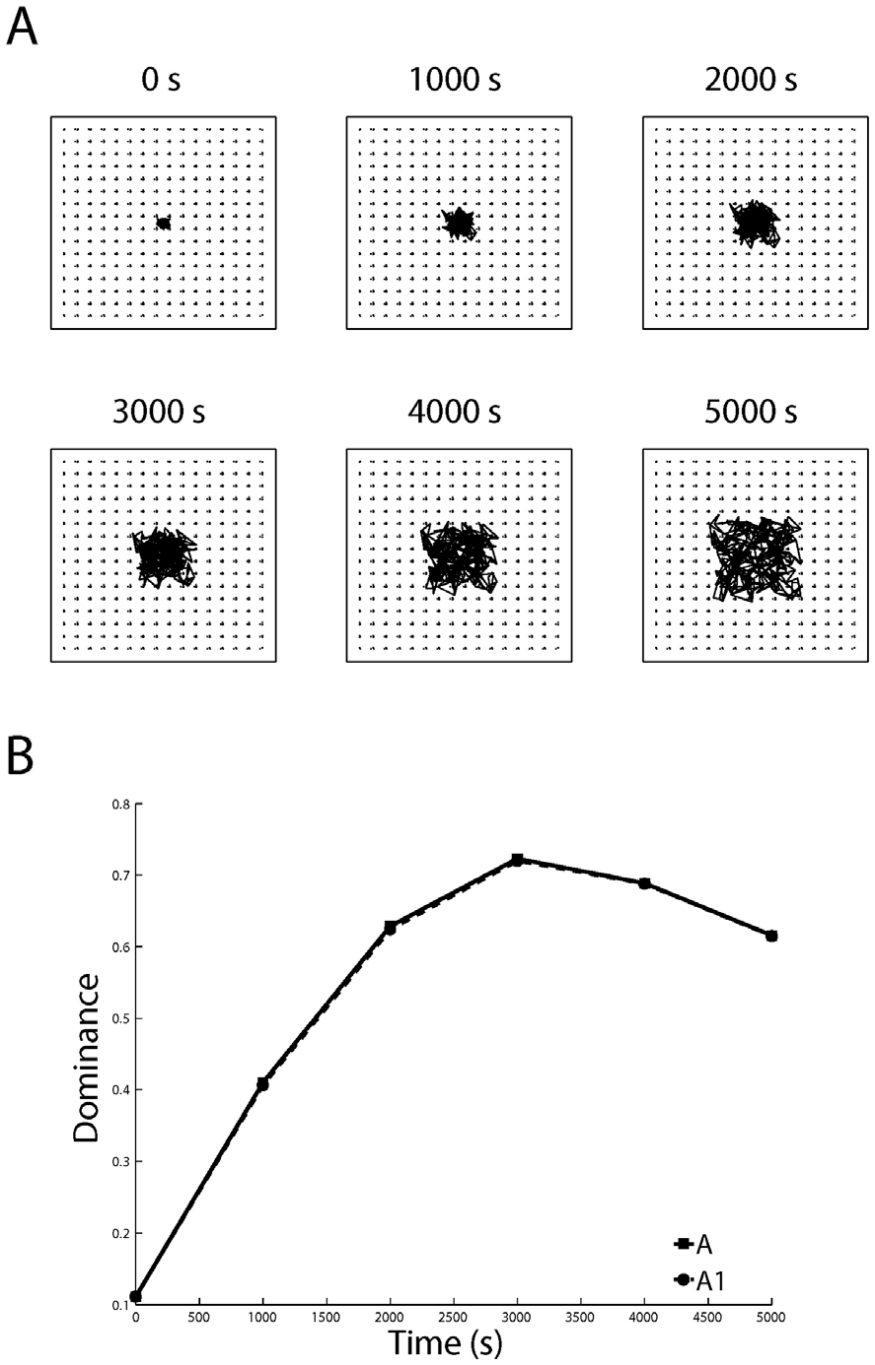
A proper topography fails to develop in the absence of structured retinal input. In these simulations, the dLGN received interleaved Poisson noise from each eye instead of retinal waves (thus preserving the anti-correlated activity between retinas). (A) The CoMs do not approach the grid as in Figures 9 and 10 (normal and accelerated waves), yet (B) the proper eye-specificity develops.

Next, to divorce layer-specificity from topography refinement, ‘random’ waves were generated by replacing the activity of RGCs with Poisson noise (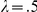), and then interleaving the noise between the two retinas, where each retina was active for one second with the other completely inactive. Intuitively, this would cause the biased retinas to dominate the appropriate layers in the dLGN since the two eyes were still anti-correlated, yet the topography would not be refined due to the lack of spatially local input. Indeed, this is what happened in the model, as DOM increased similar to the other simulations, yet the CoM for each dLGN cell did not approach its ideal position on the grid (Figure 11).

**Figure 12:**
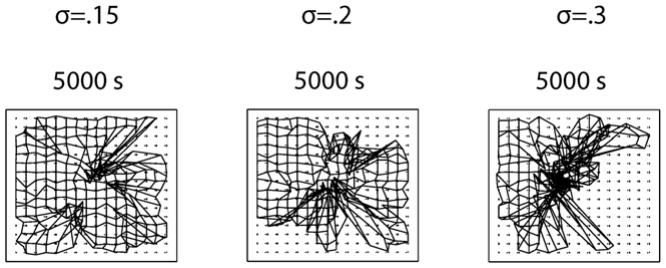
The retinogeniculate pathway still develops in the presence of waves generated by SACs with randomized refractory periods. The shape of retinal waves was altered by randomizing 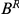 (see Figure 2), by drawing 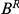 from a normal distribution centered at .09 with σ set to .15, .2 and .3 (negative values were set to 0). The final state of the model retinogeniculate projection in each case is shown. Clearly, the width of the distribution affects retinogeniculate development, with the CoMs for many dLGN cells do not fully reach their targets on the ideal grid.

Finally, waves of different geometries were simulated by randomizing the afterhyperpolarization period of the SACs by setting 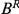 for each cell to values drawn from a Gaussian distribution centered at .09 with 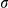 set to .15, .2 and .3 (example waves are shown in Figure 2), with negative values set to zero. A small amount of variability 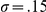 had a minimal effect on retinogeniculate development, while setting 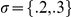 prevented full refinement of the dLGN topography, with the CoMs forming a “crumpled” pattern (Figure 12).

## Discussion

Tables 2 and 3 compare the proposed model with previous models. One innovation of the retinal dynamics in the current model is the use of a recovery variable, *r*, as in (7), to approximate the dynamics of Ca^2+^-activated and CAMP-sensitive K^+^ channels. Godfrey and Swindale [14] described a simpler model that utilizes an activity-dependent refractory period. Their model employs a countdown mechanism whereby a cell necessarily spikes after a variable decays to zero. This hypothesis does not help to explain mechanistically how the intracellular processes that underlie SAC AHPs govern wave dynamics. Another recent model of retinal waves from Hennig et al. [42] uses the idea that SACs are governed by intrinsic cellular noise and slower activity-dependent Ca^2+^ processes that dictate afterhyperpolarization periods. This is more similar to our model and is quite biophysically realistic. Our approach captures most of the dynamics present in this spiking model, yet is significantly simpler and is more amenable to embedding in a larger model of retinogeniculate development, which is not simulated by Hennig et al. More precisely, in our model, the recovery variable *r* simulates lumped intracellular inhibitory mechanisms, based on whole-cell patch clamp [24] and Ca^2+^-imaging [5] data. Similar to the role of the recovery variable in the Fitzhugh-Nagumo model of a spiking neuron, *r* suppresses the firing rate of SACs after a prolonged period of heightened firing. This approximates the process of slow Ca^2+^ accumulation in the cytosol during bursting, which progressively opens K^+^ channels that electrically shunt a given SAC. As the channels are slow to close, the SAC remains hyperpolarized or refractory for tens of seconds during the AHP, which regulates the periodicity of retinal waves observed *in vitro*. Additionally, upregulating cAMP through either uncaging techniques or bath application of forskolin reduces AHPs [5], [24], an effect simulated by the model (see Figure 4). Thus, the pacemaking mechanism of the model retina is cell-autonomous since it is regulated by intracellular processes and is effected by both Ca^2+^ and cAMP, which agrees with whole-cell patch clamp data [24] wherein SACs rhythmically depolarized even after the complete blockage of synaptic transmission.

**Table 2:** Comparision of retinogeniculate models.

Reference	Dynamical retina	Activity-dependent refractoriness	Retinogeniculate simulation
Haith, 1995			**√**
Eglen, 1999			**√**
Elliot and Shadbolt, 1999	**√**		**√**
Feller et al., 1997	**√**		
Godfrey and Swindale, 2007	**√**	**√**	
Hennig et al., 2009	**√**	**√**	
**Proposed model**	**√**	**√**	**√**

**Table 3:** Comparison of models that simulate the dLGN.

Reference	Dynamical dLGN cell simulation	Competition for neurotrophin
Haith, 1995	**√**	
Eglen, 1999	**√**	
Elliot and Shadbolt, 1999		**√**
Proposed model	**√**	**√**

This foundation enables our simulation of retinogeniculate development to use a biologically plausible dynamical system with activity-dependent refractoriness to generate retinal waves. Some models simply move the center of pre-computed distributions (e.g., a Gaussian) to approximate the bump of activity that sweeps across the retinal sheet during waves [13], [35], while others use a pre-determined refractory period that is not governed by a dynamical process [2], [34] (e.g., their model SACs are forced to not fire for a given interval after bursting). Our model also demonstrates how a simple, but biologically realistic post-synaptically gated learning rule [28] based on competition for a growth factor, as in equations (17)–(20), can simultaneously lead to eye-specificity and to the refinement of the retinogeniculate projection. That is to say, the model waves lead to LGN activity, which, in turn, induces learning where the correlations between LGN and RGC activity are the strongest. This competition obviates the need for a biologically unrealistic explicit divisive normalization term such as is used in [13], [34], [35].

In summary, the proposed model builds on and goes beyond previous work by being the first to include both: (1) a fully dynamical explanation and simulation of retinal waves that clarifies the functional role of intracellular AHP currents, and (2) a learning rule that exploits competition for a limited neurotrophin to induce topography refinement and the dominance of one eye over the other in each dLGN layer. Testing the model's predictions can provide additional experimental data with which to develop future model refinements (see Table 3).

## References

[1] Stafford BK, Sher A, Litke AM, Feldheim DA (2009). Spatial-temporal patterns of retinal waves underlying activity-dependent refinement of retinofugal projections.. Neuron.

[2] Feller MB, Butts DA, Aaron HL, Rokhsar DS, Shatz CJ (1997). Dynamic processes shape spatiotemporal properties of retinal waves.. Neuron.

[3] Meister M, Wong RO, Baylor DA, Shatz CJ (1991). Synchronous bursts of action potentials in ganglion cells of the developing mammalian retina.. Science (New York, NY).

[4] Feller MB, Wellis DP, Stellwagen D, Werblin FS, Shatz CJ (1996). Requirement for cholinergic synaptic transmission in the propagation of spontaneous retinal waves.. Science (New York, NY).

[5] Stellwagen D, Shatz CJ, Feller MB (1999). Dynamics of retinal waves are controlled by cyclic amp.. Neuron.

[6] Sernagor E, Eglen SJ, O'Donovan MJ (2000). Differential effects of acetylcholine and glutamate blockade on the spatiotemporal dynamics of retinal waves.. Journal of Neuroscience.

[7] Zhou ZJ (2001). The function of the cholinergic system in the developing mammalian retina.. Progress in Brain Research.

[8] Feller MB (2002). The role of nachr-mediated spontaneous retinal activity in visual system development.. Journal of Neurobiology.

[9] Zheng Jj, Lee S, Zhou (2004). A developmental switch in the excitability and function of the starburst network in the mammalian retina.. Neuron.

[10] Firth S, Wang C, Feller M (2005). Retinal waves: mechanisms and function in visual system development.. Cell Calcium.

[11] Blankenship AG, Ford KJ, Johnson J, Seal RP, Edwards RH (2009). Synaptic and extrasynaptic factors governing glutamatergic retinal waves.. Neuron.

[12] Burgi PY, Grzywacz NM (1994). Model for the pharmacological basis of spontaneous synchronous activity in developing retinas.. Journal of Neuroscience.

[13] Eglen SJ (1999). The role of retinal waves and synaptic normalization in retinogeniculate development.. Philosophical transactions of the Royal Society of London Series B, Biological sciences.

[14] Godfrey KB, Swindale NV (2007). Retinal wave behavior through activity-dependent refractory periods.. PLoS Comput Biol.

[15] Sun C, Warland DK, Ballesteros JM, van der List D, Chalupa LM (2008). Retinal waves in mice lacking the β2 subunit of the nicotinic acetylcholine receptor.. Proceedings of the National Academy of Sciences of the United States of America.

[16] Torborg C, Feller M (2005). Spontaneous patterned retinal activity and the refinement of retinal projections.. Progress in Neurobiology.

[17] Chapman B (2000). Necessity for afferent activity to maintain eye-specific segregation in ferret lateral geniculate nucleus.. Science.

[18] Huberman AD, Speer CM, Chapman B (2006). Spontaneous retinal activity mediates development of ocular dominance columns and binocular receptive fields in v1.. Neuron.

[19] Bansal A, Singer JH, Hwang BJ, Xu W, Beaudet A (2000). Mice lacking specific nicotinic acetylcholine receptor subunits exhibit dramatically altered spontaneous activity patterns and reveal a limited role for retinal waves in forming on and off circuits in the inner retina.. Journal of Neuroscience.

[20] Cherubini E, Gaiarsa J, Ben-Ari Y (1991). GABA: an excitatory transmitter in early postnatal life.. Trends in Neurosciences.

[21] Sah P (2002). Channels underlying neuronal calcium-activated potassium currents.. Progress in Neurobiology.

[22] Dunn T, Wang C, Colicos M, Zaccolo M, DiPilato L (2006). Imaging of cAMP levels and protein kinase A activity reveals that retinal waves drive oscillations in second-messenger cascades.. Journal of Neuroscience.

[23] Fitzhugh R (1961). Impulses and physiological states in theoretical models of nerve membrane.. Biophysical Journal.

[24] Zheng J, Lee S, Zhou ZJ (2006). A transient network of intrinsically bursting starburst cells underlies the generation of retinal waves.. Nature Neuroscience.

[25] Udin SB, Fawcett JW (1988). Formation of topographic maps.. Annual Review of Neuroscience.

[26] Sretavan DW, Shatz CJ (1987). Axon trajectories and pattern of terminal arborization during the prenatal development of the cat's retinogeniculate pathway.. Journal of Comparative Neurology.

[27] Shatz CJ (1983). The prenatal development of the cat's retinogeniculate pathway.. Journal of Neuroscience.

[28] Grossberg S, Seitz A (2003). Laminar development of receptive fields, maps and columns in visual cortex: the coordinating role of the subplate.. Cerebral Cortex.

[29] Grossberg S, Williamson JR (2001). A neural model of how horizontal and interlaminar connections of visual cortex develop into adult circuits that carry out perceptual grouping and learning.. Cerebral Cortex.

[30] Grossberg S, Williamson JR (1999). A self-organizing neural system for learning to recognize textured scenes.. Vision Research.

[31] Riddle DR, Lo DC, Katz LC (1995). Nt-4-mediated rescue of lateral geniculate neurons from effects of monocular deprivation.. Nature.

[32] Bonhoeffer T (1996). Neurotrophins and activity-dependent development of the neocortex.. Current Opinion in Neurobiology.

[33] Mcallister KA, Katz LC, Lo DC (1996). Neurotrophin regulation of cortical dendritic growth requires activity.. Neuron.

[34] Elliott T, Shadbolt NR (1999). A neurotrophic model of the development of the retinogeniculocortical pathway induced by spontaneous retinal waves.. Journal of Neuroscience.

[35] Haith GL (1998). Modeling activity-dependent development in the retinogeniculate projection.. http://portal.acm.org/citation.cfm?id=927417.

[36] Beggs JM, Plenz D (2003). Neuronal avalanches in neocortical circuits.. Journal of Neuroscience.

[37] Huberman AD, Wang GY, Liets LC, Collins OA, Chapman B (2003). Eye-specific retinogeniculate segregation independent of normal neuronal activity.. Science.

[38] Huberman A (2007). Mechanisms of eye-specific visual circuit development.. Current Opinion in Neurobiology.

[39] Oleary D, Mclaughlin T (2005). http://dolearysalk.edu.

[40] Sretavan DW, Shatz CJ, Stryker MP (1988). Modification of retinal ganglion cell axon morphology by prenatal infusion of tetrodotoxin.. Nature.

[41] Wong ROL (1999). Retinal waves and visual system development.. Annual Review of Neuroscience.

[42] Hennig M, Adams C, Willshaw D, Sernagor E (2009). Early-stage waves in the retinal network emerge close to a critical state transition between local and global functional connectivity.. The Journal of Neuroscience.

